# Genetic testing in chronic kidney disease of uneXplained cause (CKDx): clinical insights and evolving diagnostic paradigms

**DOI:** 10.1515/medgen-2025-2046

**Published:** 2026-02-18

**Authors:** Jan Halbritter, Matias Simons

**Affiliations:** Charité – Universitätsmedizin Berlin Department of Nephrology and Medical Intensive Care Charitéplatz 1 10117 Berlin Germany; Heidelberg University Hospital Institute of Human Genetics Im Neuenheimer Feld 366 69120 Heidelberg Germany

## Abstract

Chronic kidney disease (CKD) represents a significant global health burden, with diverse etiologies and often complex clinical presentations. Among these, a notable subset of CKD patients present without a clear underlying cause despite extensive diagnostic evaluation. For this subgroup, the term CKDx – chronic kidney disease of unexplained cause, has recently been proposed. A major element of the diagnostic workup of CKDx is genetic testing, for which the methodology has greatly improved in the last years.

## The emergence of genetic testing in CKDx

CKD is a progressive condition characterized by the gradual loss of kidney function over time, affecting an estimated 10–15 % of the global adult population. It encompasses a wide range of underlying etiologies or contributing factors, including diabetes, hypertension, glomerular diseases, and genetic disorders. Historically, kidney biopsies and histopathology have been central in diagnosing CKD causes, guiding therapy and prognosis. However, despite detailed histological workups, many cases remain labeled as nonspecific or idiopathic, which complicates management and patient counseling. For this situation, the term CKDx (chronic kidney disease of uneXplained cause) was recently suggested by the Genes & Kidney working group of the European Renal Association [1].

The landscape of diagnosing CKD has been profoundly transformed by advances in genetic testing, particularly through the use of exome sequencing (ES). Prior to these developments, many cases of CKD were classified as idiopathic or attributed to non-genetic causes, often leaving patients and clinicians without definitive answers. This changed dramatically with the publication of a landmark study in 2019 [2], which applied ES to a large cohort of predominantly adult CKD patients to detect almost 10 % of the study population being affected by an inherited form. Interestingly, the authors found the diagnostic yield to be highest in the subgroup of unexplained CKD, exceeding 15 %. This study was groundbreaking, as it revealed that a significant proportion of patients previously thought to have sporadic or environmentally-driven disease actually harbored monogenic causes identifiable through comprehensive genetic analysis. Importantly, this genetic diagnosis often had immediate clinical implications, influencing patient management, family counseling, and transplant considerations. This work fundamentally challenged the prevailing assumption that genetic kidney diseases, especially in adults, were rare and limited to patients with clear family histories or syndromic manifestations.

Following this pioneering effort, several subsequent studies reinforced and extended these findings, by either focusing on the pre-transplant population [3–5] or unexplained etiologies in national cohorts [6]. One such study [7] conducted a similarly comprehensive genetics-first analysis in adults with CKD onset before the age of 60. This research demonstrated that between 15 % and 25 % of patients received a definitive genetic diagnosis, with diagnostic yield closely linked to factors such as early age at disease onset, presence of extrarenal manifestations, and family history of kidney disease. Notably, even patients without obvious syndromic features or known affected relatives benefited from genetic testing, highlighting the often-subclinical presentation of genetic kidney diseases. Another important contribution came from a recent Australian study [8], which applied whole genome sequencing to a diverse CKD cohort and reported diagnostic yields on the higher end of this range, approaching 25 %. Collectively, these studies demonstrate that genetic etiologies underlie a substantial fraction of CKD cases previously classified as idiopathic, suggesting that monogenic kidney diseases are not rare outliers but a significant and clinically relevant subgroup.

**Figure 1:**
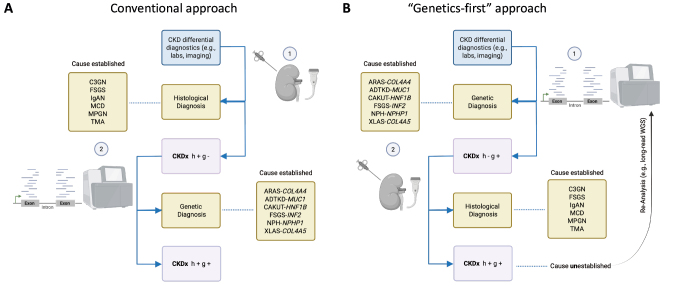
Schematic on diagnostic strategies for identification of the underlying cause in a patient with CKD. **(A)** Conventionally, kidney biopsy is the gold standard for establishing the underlying kidney disorder by providing clinicians with a histological diagnosis. In cause of undetermined findings upon histology (CKDx h+ g-), genetic testing serves as an 2^nd^ tier diagnostic tool to establish the definite cause. In cases of unestablished etiology despite histological and genetic investigation, the term CKDx with the suffices h+ and g+ has been proposed. **(B)** In a “Genetics-first” approach, genetic testing is considered the 1^st^ tier diagnostics for establishing the underlying cause. In unresolved cases (CKDx h+ g+), re-analysis, for instance by long-read sequencing, will be indicated (Figure created with Biorender). Legend: ADPKD – autosomal dominant polycystic kidney disease; ADTKD – autosomal dominant tubulointerstitial kidney disease; ARAS – autosomal recessive Alport Syndrome; CAKUT – congenital anomalies of the kidneys and urinary tract; CKDx – chronic kidney disease of unexplained cause; C3GN – Complement factor 3 glomerulonephritis; FSGS – focal segmental glomerulosclerosis; IgAN – IgA Nephropathy; MCD – minimal change disease; MPGN – membranoproliferative glomerulonephritis; NPH – nephronophthisis; TMA – thrombotic microangiopathy.

In response to this growing body of evidence, there is increasing advocacy for the integration of genetic testing early in the diagnostic algorithm for CKD rather than reserving it as a last step after exhaustive clinical and histopathological evaluation (**Figure 1**) [1, 9] Although renal histology remains the diagnostic gold-standard, early genetic diagnosis may prevent unnecessary biopsies, avoid ineffective treatments, and expedite access to appropriate clinical trials or targeted therapies. Additionally, the identification of a genetic cause facilitates cascade testing of family members, allowing early detection and preventive care in at-risk relatives. Lastly, studies have also demonstrated cost-effectiveness, showing a 20–40 % cost reduction through diagnostic workflows that incorporate early genetic testing, leading to reduced hospitalizations and lower rates of biopsy-related complications [10]. Importantly, the emergence of ES and related genomic technologies represents a paradigm shift in nephrology that aligns with the broader move towards precision medicine, where comprehensive genetic insights enable tailored and more effective management of CKD. Finally, genetics and histology are complementary, jointly advancing diagnostic accuracy. Therefore, CKDx was proposed with the suffixes “h” and “g” to indicate whether renal histology and genetic testing have been conducted (“+/-“), respectively (**Figure 1**).

## Genetic kidney diseases underlying CKDx

The spectrum of genetic kidney diseases contributing to CKDx is broad and heterogeneous. These conditions range from well-characterized monogenic disorders to newly discovered genes with variable penetrance and expressivity.

One prominent example is Alport syndrome, a hereditary nephropathy caused by pathogenic variants in genes encoding type IV collagen chains (*COL4A3, COL4A4*, and *COL4A5*). Traditionally, Alport syndrome is associated with hematuria, progressive proteinuria, sensorineural hearing loss, and ocular anomalies. However, the expressivity is highly variable. Many patients with Alport variants do not present with classic extrarenal features and instead manifest isolated CKD or atypical histopathology, often leading to misclassification under nonspecific glomerular diseases such as focal segmental glomerulosclerosis (FSGS). This variability complicates clinical recognition and highlights the critical role of genetic testing in uncovering cryptic Alport syndrome within CKDx populations. Investigations using population databases, such as gnomAD, have revealed that the frequency of heterozygous pathogenic variants in *COL4A3* or *COL4A4* is approximately 1 in 100 in the general population [11]. Given the incomplete penetrance in many cases, this discovery has led to the recognition of heterozygous pathogenic variants in *COL4A3* and *COL4A4* as CKD risk alleles. Furthermore, in the X-chromosomal gene *COL4A5*, pathogenic founder variants such as p.Gly624Asp have been associated with milder clinical courses, which probably facilitated their wider occurrence through evolution [12]. While this high prevalence supports genetic testing, it also underscores that genetics alone may not capture the full diagnostic picture. In some cases, histological assessment remains essential to avoid missing coexisting entities – such as IgA nephropathy or other glomerular diseases – that may account for both interfamilial and intrafamilial variability.

Other genetic causes include autosomal dominant tubulointerstitial kidney disease (ADTKD), caused by pathogenic variants in genes like *UMOD* and *MUC1*, which can present with slowly progressive CKD and nonspecific biopsy findings. ADTKD often evades diagnosis due to subtle or absent family history and overlapping clinical features with acquired CKD. Inherited glomerulopathies beyond Alport syndrome, including pathogenic variants in genes associated with nephrotic syndrome or complement-mediated diseases such as atypical hemolytic uremic syndrome (aHUS) also contribute to CKDx. The final example is defects in *NPHP1*, which represent the most common cause of the renal ciliopathy nephronophthisis (NPH). In contrast to its traditional recognition as a pediatric form of CKD, recent studies have shown that biallelic sequence alterations in *NPHP1* account for more than 0.5 % of adult kidney failure cases [13].

## Implementing genetic testing: strategies and considerations

The integration of genetic testing into nephrology care involves navigating complex logistical, interpretative, and ethical dimensions. Moreover, the choice of testing modality – gene panels, ES, or genome sequencing (GS) – affects diagnostic yield, cost, and downstream clinical decision-making.

Gene panels targeting known kidney disease genes are widely used and can be generated either through targeted capture or by applying virtual panels to ES/GS data. Current curated panels include over 600 genes implicated in renal disorders. These panels provide a focused approach, limiting incidental findings but potentially missing novel or unexpected genes and phenotypic expansion of monogenic diseases.

ES, which sequences all protein-coding regions of the genome, has become the cornerstone in CKDx diagnostics due to its comprehensive coverage and cost-effectiveness. It offers higher diagnostic yield than limited panels and captures a broad range of pathogenic variants, including single nucleotide variants and small insertions/deletions. However, ES does not reliably detect copy number variants (CNVs), structural rearrangements, or noncoding variations, which may necessitate supplementary testing.

As genetic testing becomes more central to diagnosing CKDx, the limitations of current methods have driven the exploration and adoption of increasingly comprehensive sequencing technologies. This gap has spurred the use of GS, which offers the most extensive genomic coverage currently available. GS sequences the entire genome, encompassing coding and noncoding DNA, regulatory elements, introns, and mitochondrial DNA. This comprehensive approach increases the likelihood of detecting variants that ES and targeted panels may miss, including CNVs, large deletions or duplications, and deep intronic variants that can disrupt gene function. For example, certain pathogenic variants in genes causing kidney disease may lie in regulatory regions or affect splicing, which ES cannot reliably capture. GS also enables more uniform coverage of complex genes that pose challenges for capture-based techniques. One such challenge is exemplified by ***PKD1***, the major culprit in autosomal dominant polycystic kidney disease (ADPKD). *PKD1* is notoriously difficult to sequence accurately because it has six highly homologous pseudogenes that share about 97 % sequence identity. These pseudogenes are scattered near *PKD1* on chromosome 16 and can lead to misalignment and false variant calls when using capture-based sequencing techniques like ES [14].

Altogether, GS provides more comprehensive data and can improve resolution in such problematic genomic regions, although even GS has technical challenges here due to sequence similarity. This is where emerging technologies such as long-read sequencing hold particular promise. Long-read sequencing platforms, such as Oxford Nanopore Technologies and Pacific Biosciences (PacBio), generate reads thousands to tens of thousands of base pairs in length, far exceeding the typical 100–150 base pair reads of short-read sequencing. These longer reads allow for more accurate mapping across repetitive regions, structural variants, and complex genomic architectures. Long-read sequencing is especially powerful for detecting genetic alterations that are refractory to short-read approaches, including large insertions or deletions, copy number changes, and tandem repeat expansions. *MUC1*, for example, harbors a variable number tandem repeat (VNTR) region that is highly repetitive and structurally complex. The pathogenic variant responsible for ADTKD-*MUC1* involves a single cytosine duplication within this VNTR, which is essentially invisible to standard short-read sequencing technologies. Specialized approaches, including long-read sequencing or bioinformatics tools like VNtyper, are needed to detect these variants reliably [15–17]. Furthermore, long-read sequencing enhances detection of structural variants within gene clusters relevant to kidney disease. For example, atypical hemolytic uremic syndrome (aHUS) can involve complex rearrangements within the *CFHR* gene cluster, which are difficult to resolve with short reads but can be characterized using long-read platforms (**Table 1**).

Although these advanced sequencing methods are not yet routine in clinical nephrogenetic diagnostics due to cost, technical complexity, and bioinformatic challenges, their rapid technological improvements and decreasing costs suggest they will become increasingly integrated into diagnostic workflows. Their implementation promises to substantially increase diagnostic yields, especially in difficult cases where prior ES and gene panel testing were inconclusive.

## The essential role of deep clinical phenotyping in genetic testing for CKDx

Accurate clinical phenotyping remains, nonetheless, a cornerstone throughout the entire genetic testing process for CKDx. Genetic sequencing alone, whether through targeted panels, ES, or GS, cannot fully substitute for detailed clinical information. Instead, clinical data provide essential context that guides the interpretation of genetic variants and helps distinguish causal variants from benign variants or incidental findings.

In recent years, the concept of deep phenotyping has gained traction as a systematic approach to collecting and encoding detailed patient data. Deep phenotyping involves the comprehensive and precise characterization of clinical signs, symptoms, laboratory findings, histopathological features, and radiological data. This approach goes far beyond standard medical history taking and involves standardized vocabularies and ontologies to translate complex clinical presentations into structured data that can be utilized in computational analyses.

One of the most powerful tools facilitating deep phenotyping is the Human Phenome Ontology (HPO), an internationally accepted standardized vocabulary of phenotypic abnormalities encountered in human disease [18]. By mapping patient features to HPO terms, clinicians and geneticists can create a machine-readable profile of the phenotype, which can then be used to prioritize candidate genes and variants in bioinformatic pipelines. This standardized language allows better integration and comparison across patient datasets and improves the sensitivity and specificity of variant filtering algorithms.

For example, a patient presenting with proteinuria, sensorineural hearing loss, and microscopic hematuria could be assigned specific HPO terms corresponding to each symptom. When genetic data are analyzed with these phenotypic tags, variants in genes known to cause syndromes that match the phenotype – such as *COL4A3, COL4A4*, *COL4A5* in Alport syndrome, or *MYH9* in Alport syndrome-like – can be prioritized for further evaluation. Importantly, this approach may help draw attention to genes often overlooked by clinicians, such as *MT-TL1*, a mitochondrial DNA gene that, when mutated, can cause disorders like MELAS (mitochondrial encephalopathy, lactic acidosis, and stroke-like episodes). This targeted approach reduces noise in sequencing data, highlighting variants more likely to explain the clinical picture.

An equally important practice linked to deep phenotyping is reverse phenotyping, which entails re-examining and refining clinical features in light of genetic findings. Often, initial clinical assessments may overlook subtle or atypical manifestations of a genetic condition, particularly in diseases like CKDx where presentations can be nonspecific or variable. After identifying a variant of interest, clinicians can revisit the patient’s history, physical examination, and diagnostic workup to confirm or refute the suspected diagnosis.

Reverse phenotyping helps in several ways [19, 20]. First, it can validate the pathogenicity of variants that might otherwise be classified as variants of uncertain significance (VUS). By identifying overlooked signs consistent with the genetic diagnosis, clinicians and geneticists gain confidence that the variant is truly causal. For instance, a patient with an uncertain *COL4A5* variant might undergo audiometry and eye examinations after genetic results, revealing subtle hearing loss or ocular changes typical of Alport syndrome, thus confirming the diagnosis. Conversely, absence of supportive clinical features may prompt reconsideration of the variant’s role or encourage further testing. Second, reverse phenotyping mitigates misdiagnosis and inappropriate management. Misinterpreting a genetic variant without proper clinical correlation risks labeling patients incorrectly, potentially leading to unnecessary or harmful interventions. For example, a misclassified pathogenic variant in a gene unrelated to the patient’s phenotype could result in unwarranted immunosuppressive therapy or incorrect prognostic counseling.

**Table 1:** Complex genetic loci associated with inherited kidney disease

Locus	Genes (reasons for complexity)	Disease(s)	Diagnostics
Chr. 1q32; RCA (Regulation of Complement Activation)	*CFH*, *CFHR1-CFHR5 (CFH-CFHR hybrid genes).*	aHUS, C3GN	Customized panels / Long-read sequencing
Chr. 1q22	*MUC1* *(VNTR – variable number tandem repeat*)	ADTKD	VNtyper^15^ / Long-read sequencing
Chr. 16p13.3	*PKD1 (pseudogene region exon 1–32)*	ADPKD	Customized panels^35^ / Long-read sequencing

Legend: aHUS – atypical hemolytic uremic syndrome, ADTKD – autosomal dominant tubulointerstitial kidney disease, ADPKD – autosomal dominant polycystic kidney disease, C3GN – complement factor 3 glomerulonephritis.

Moreover, deep phenotyping enables identification of phenotypic expansions – situations where known genetic disorders manifest with atypical or broader clinical features than previously described. In nephrogenetics, many syndromes show considerable variability, and expanding the phenotypic spectrum can improve recognition and diagnosis. This paradigmatically applies to congenital anomalies of the kidney and urinary tract (CAKUT) where impaired organ involvement and consecutive clinical presentations, even upon the same genetic aberration, are extremely heterogeneous [21, 22]. Deep phenotyping can capture such nuances and allow for more sensitive genetic interpretation. Practically, the integration of deep phenotyping into clinical workflows requires multidisciplinary collaboration between nephrologists, geneticists, pathologists, and bioinformaticians. Digital tools (e.g., SAMS – *Symptom annotation made simple* [23]) and electronic health records can facilitate standardized data capture, but clinical expertise remains essential to ensure accurate and comprehensive data entry. The dynamic interplay between detailed clinical assessment and genetic data analysis exemplifies the future of precision nephrology, where diagnosis and management are tailored to the individual’s unique genetic and phenotypic profile.

## Interpreting genetic results: challenges and best practices

Genetic test interpretation requires careful integration of molecular findings with clinical context. Variants are classified according to American College of Medical Genetics and Genomics (ACMG) guidelines into five categories: pathogenic, likely pathogenic, variant of uncertain significance (VUS), likely benign, and benign [24]. While pathogenic and likely pathogenic variants typically confirm diagnosis, they must be interpreted cautiously, especially in cases of incomplete penetrance or atypical inheritance patterns.

VUS are a common outcome in CKDx testing and pose a challenge. These variants are reported only when there is reasonable suspicion of pathogenicity and a potential pathway for future reclassification (https://www.acgs.uk.com/quality/best-practice-guidelines/). Periodic reanalysis, ideally every two to three years, is recommended as new evidence emerges. Collaborative multidisciplinary discussions, involving nephrologists, geneticists, and genetic counselors, are essential to contextualize findings, decide on further functional testing, and guide clinical management.

Negative or inconclusive genetic results do not exclude a genetic basis. Limitations of testing platforms, gene content, and knowledge gaps mean that some pathogenic variants remain undetected or unclassified. As highlighted above, the detection of VNTR aberrations in *MUC1* requires specific methods beyond standard sequencing. Furthermore, the absence of CNV analysis can miss clinically relevant deletions or duplications. Clinicians should remain vigilant, consider re-testing as technologies evolve, and involve centers of expertise to bridge diagnostic gaps – for instance, through one of the 72 centers comprising the European Rare Kidney Disease Reference Network across 24 EU member states [25].

## Clinical and familial implications of genetic diagnosis

A confirmed genetic diagnosis has profound clinical implications. It guides personalized patient care by enabling tailored therapies, avoiding ineffective or harmful treatments, and informing prognosis [9]. For instance, recognizing a genetic form of FSGS might preclude unnecessary immunosuppressive therapies, such as long-term administration of steroids or nephrotoxic calcineurin inhibitors. On the other hand, genetic diagnoses may also enable targeted treatment options, exemplified by licensed RNAi-based therapies (e.g., lumasiran for primary hyperoxaluria type 1 [26]) or pharmacological chaperones (e.g., migalastat for Fabry disease [27]). Genetic findings also impact family counseling, reproductive decision-making, and cascade testing of relatives for early diagnosis or organ donor suitability. In the context of kidney transplantation, genetic diagnoses provide information about the risk of disease recurrence in the allograft. While in most inherited kidney diseases the structural defect is confined to the native kidneys, making recurrence unlikely, there are conditions with a high likelihood of recurrence, for which preventive measures are essential to avoid graft loss. Examples include conditions involving disturbed autoimmunity (e.g., complement-related kidney diseases / aHUS [28]), enzymatic defects leading to renal crystal deposition (e.g., primary hyperoxalurias [29], adenine phosphoribosyltransferase deficiency [14]), and metabolic disorders of glucose metabolism (e.g., Maturity-Onset Diabetes of the Young [30]).

Specific alleles such as the *APOL1* G1 and G2 risk variants, highly prevalent in individuals of West African ancestry, exemplify the interplay between genetics and environment in CKD risk [31]. Recently, it has been found that harboring the additional *APOL1* missense variant (p.N264K) mitigates pathogenicity in carriers of the G2 risk variant and therefore serves as a strongly protective intragenic modifier [32]. Including *APOL1* in genetic panels is crucial given its clinical relevance, particularly in patients with proteinuria or biopsy-proven FSGS. Early diagnosis is particularly important in light of emerging disease-specific treatment options, such as inaxaplin for APOL1-mediated kidney disease [33].

## Outlook: towards precision nephrology

The field of nephrogenetics is rapidly evolving. Integration of comprehensive genetic testing with clinical, histopathological, and biomarker data promises to redefine CKDx classification and management. Emerging technologies like GS and long-read sequencing are poised to increase diagnostic yields, uncover novel disease mechanisms, and enable early, cause-directed interventions, as exemplified by the rising number of targeted gene- and cell-based therapies in preclinical and clinical trials (e.g., microRNA-17 inhibitor farabursen for ADPKD, NCT05521191) [34].

Efforts to establish registries for CKDx patients and harmonize diagnostic codes are underway, facilitating research, data sharing, and standardized care pathways. Multidisciplinary nephrogenetic teams and centers of expertise play pivotal roles in providing high-quality diagnostic services and ongoing education.

In summary, genetic testing has emerged as a cornerstone in unraveling the mysteries of CKDx, with current diagnostic yields of 15–25 % in appropriately selected patients. Continuous technological advances and growing clinical experience will further enhance precision diagnostics, transforming care for patients with chronic kidney disease.
